# The Arabidopsis immune regulator *SRFR* *1* dampens defences against herbivory by *S* *podoptera exigua* and parasitism by *H* *eterodera schachtii*

**DOI:** 10.1111/mpp.12304

**Published:** 2015-11-06

**Authors:** Phuong Dung T. Nguyen, Sharon Pike, Jianying Wang, Arati Nepal Poudel, Robert Heinz, Jack C. Schultz, Abraham J. Koo, Melissa G. Mitchum, Heidi M. Appel, Walter Gassmann

**Affiliations:** ^1^ Division of Plant Sciences and Interdisciplinary Plant Group University of Missouri Columbia MO 65211‐7310 USA; ^2^ Christopher S. Bond Life Sciences Center University of Missouri Columbia MO 65211‐7310 USA; ^3^ Division of Biochemistry and Interdisciplinary Plant Group University of Missouri Columbia MO 65211‐7310 USA; ^4^ Present address: Department of Biology Denison University Granville OH 43023 USA

**Keywords:** *A**rabidopsis thaliana*, beet armyworm, beet cyst nematode, jasmonic acid, salicylic acid, signalling

## Abstract

Plants have developed diverse mechanisms to fine tune defence responses to different types of enemy. Cross‐regulation between signalling pathways may allow the prioritization of one response over another. Previously, we identified *SUPPRESSOR OF rps4‐RLD* *1* (*SRFR* *1*) as a negative regulator of *ENHANCED DISEASE SUSCEPTIBILITY1* (*EDS1*)‐dependent effector‐triggered immunity against the bacterial pathogen *P* *seudomonas syringae* pv. tomato strain DC3000 expressing *avr* *R* *ps4*. The use of multiple stresses is a powerful tool to further define gene function. Here, we examined whether *SRFR* *1* also impacts resistance to a herbivorous insect in leaves and to a cyst nematode in roots. Interestingly, *srfr1‐1* plants showed increased resistance to herbivory by the beet army worm *S* *podoptera exigua* and to parasitism by the cyst nematode *H* *eterodera schachtii* compared with the corresponding wild‐type Arabidopsis accession RLD. Using quantitative real‐time PCR (qRT‐PCR) to measure the transcript levels of salicylic acid (SA) and jasmonate/ethylene (JA/ET) pathway genes, we found that enhanced resistance of *srfr1‐1* plants to *S*. *exigua* correlated with specific upregulation of the MYC2 branch of the JA pathway concurrent with suppression of the SA pathway. In contrast, the greater susceptibility of RLD was accompanied by simultaneously increased transcript levels of SA, JA and JA/ET signalling pathway genes. Surprisingly, mutation of either *SRFR* *1* or *EDS* *1* increased resistance to *H* *. schachtii*, indicating that the concurrent presence of both wild‐type genes promotes susceptibility. This finding suggests a novel form of resistance in Arabidopsis to the biotrophic pathogen *H* *. schachtii* or a root‐specific regulation of the SA pathway by EDS1, and places *SRFR* *1* at an intersection between multiple defence pathways.

## Introduction

A variety of perception mechanisms, inducible signal cascades and transcriptional regulation of many defence‐ and stress‐related genes enable plants to survive and reproduce in challenging environments (Van Poecke, 2007). In general, salicylic acid (SA)‐dependent responses are triggered by and active against biotrophic pathogens, whereas resistance to necrotrophs and insect herbivores is usually associated with the jasmonic acid (JA)/ethylene (ET) pathway. Predominantly, these two defence pathways have been observed to act antagonistically, which allows plants to prioritize their response to a prevailing threat (Glazebrook, 2005; Kunkel and Brooks, 2002; Pieterse *et al*., 2012; Zander *et al*., 2014). However, in a network analysis, both pathways contributed to resistance to biotrophs and necrotrophs (Tsuda *et al*., 2009), consistent with considerable overlap in the transcriptional response to these hormones (Schenk *et al*., 2000) and hormone concentration‐dependent SA and JA/ET synergism or antagonism in plant resistance (Mur *et al*., 2006). Although these responses have been studied extensively in shoots, less is known about the contribution of these pathways to the resistance of roots to pests and pathogens.

The Arabidopsis accession RLD is defective in *RPS4* and is fully susceptible to DC3000 and DC3000 carrying the effector *avrRps4* (Gassmann *et al*., 1999; Hinsch and Staskawicz, 1996). Mutations in *SUPPRESSOR OF rps4‐RLD1* (*SRFR1*) in RLD or combined with a mutation in the *RPS6* resistance gene in the RLD background result in resistance to DC3000(*avrRps4*) or DC3000(*hopA1*), respectively, whereas mutants remain fully susceptible to DC3000 (Kim *et al*., 2009b; Kwon *et al*., 2009). Specific enhancement of these effector‐triggered immunity pathways in *srfr1* mutants is most likely a consequence of SRFR1 interaction with the positive immune regulator ENHANCED DISEASE SUSCEPTIBILITY1 (EDS1) (Bhattacharjee *et al*., 2011), which, in turn, was found to be guarded by the Toll/interleukin‐1 receptor–nucleotide binding–leucine‐rich repeat (TNL) resistance proteins RPS4 and RPS6, and targeted by the effectors AvrRps4 and HopA1 (Bhattacharjee *et al*., 2011; Heidrich *et al*., 2011). Recently, the RPS4 co‐resistance protein RRS1 (Birker *et al*., 2009; Narusaka *et al*., 2009, 2013) has been identified as an additional target of AvrRps4 (Sarris *et al*., 2015; Williams *et al*., 2014), although it is not yet clear whether this interaction is direct or mediated by a common factor. Like EDS1, SRFR1 may have additional functions beyond specific effector‐triggered immunity pathways. To address this question, we used multiple stresses as a valuable tool to further elucidate gene function.

The recessive *srfr1* phenotype and the similarity of SRFR1 to transcriptional repressors of *Saccharomyces cerevisiae* and *Caenorhabditis elegans* led to the hypothesis that it functions as a negative transcriptional regulator (Kwon *et al*., 2009), which is consistent with the observed upregulation of defence genes in *srfr1* plants (Kim *et al*., 2009a) and interaction with TEOSINTE BRANCHED1/CYCLOIDEA/PCF (TCP) transcription factors (Kim *et al*., 2014). The upregulation of both SA and JA/ET defence pathway genes in *srfr1* mutants suggests that SRFR1 may also influence resistance in Arabidopsis to pests and pathogens, such as the sugar beet cyst nematode *Heterodera schachtii* and the beet army worm *Spodoptera exigua*.

The chewing insect *S. exigua* is of significant agricultural interest and feeds on the leaves of more than 50 plant species worldwide, including many crops and vegetables (Smits *et al*., 1987). When its larvae feed on plants, wounding and probably also larvae‐derived elicitors trigger the biosynthesis of JA, ET and JA derivatives, leading to defence activation (Bonaventure *et al*., 2011; Wu and Baldwin, 2010). However, it has also been reported that *S. exigua* can manipulate plant defences to activate the SA pathway and counteract the JA/ET pathway (Diezel *et al*., 2009).

*Heterodera schachtii*, a sedentary, obligate endoparasite, also infects many economically important crops, including sugar beet, cabbage and broccoli, and causes extensive worldwide agricultural and horticultural crop losses (Evans and Rowe, 1998). Second‐stage infective juveniles (J2) hatch from eggs in the soil and infect the roots of host plants using a stylet to puncture the cell wall and to secrete enzymes that degrade plant cell walls and effectors that modify host cells and processes (Hewezi and Baum, 2013; Mitchum *et al*., 2013). The fusing of the initial feeding cell with neighbouring cells forms a syncytium that feeds the sedentary nematode as it develops into a reproductive male or female adult. Adult males leave the root to mate with sedentary females exposed on the root surface. The dead female body becomes the cyst which protects the eggs until conditions are favourable for hatching.

Although recent advances have been made in the identification of nematode effectors and corresponding resistance genes (Cook *et al*., 2012; Hewezi and Baum, 2013; Liu *et al*., 2012; Mitchum *et al*., 2013), plant resistance mechanisms that limit nematode reproduction are not well understood (Kandoth and Mitchum, 2013). *Heterodera schachtii* parasitism upregulates *PR1* (*PATHOGENESIS‐RELATED GENE 1*) expression in shoots and roots of infected Arabidopsis, and overexpression of *PR1* reduces susceptibility of Arabidopsis to *H. schachtii* (Hamamouch *et al*., 2011; Wubben *et al*., 2008). The JA pathway has been reported to be important in inducing resistance to root‐knot nematode infection in rice (Cooper *et al*., 2005; Nahar *et al*., 2011); however, a tomato mutant impaired in JA perception is more resistant (Bhattarai *et al*., 2008).

Here, we show in feeding experiments that the Arabidopsis *srfr1‐1* mutant exhibits significantly enhanced resistance to *S. exigua* feeding and *H. schachtii* parasitism when compared with RLD in leaves and roots, respectively. We further present evidence that constitutive JA, JA‐Ile (JA conjugated with the amino acid isoleucine) and SA levels are not responsible for these differences. In insect experiments, higher transcript levels of both SA and JA pathway genes in mock‐treated *srfr1‐1* leaves than in RLD indicate a low level of generalized constitutive defences. Following insect herbivory, concurrent induction of the JA pathway and repression of the JA/ET and SA pathways in *srfr1‐1* is consistent with the finding that the JA pathway is mainly responsible for insect resistance in plants (Wasternack and Hause, 2013). In contrast, simultaneously increased levels of transcripts in all three pathways following insect herbivory appear to correlate with the greater susceptibility of RLD. In nematode‐parasitized roots, neither activation of the SA nor JA pathway appears to correlate with greater *srfr1‐1* resistance. Instead, increased levels of *EDS1* transcript correlate with RLD susceptibility. Correspondingly, we showed that *eds1‐2* mutants were more resistant than wild‐type Col‐0 to *H. schachtii* infection. Mutations in *PAD4* (*PHYTOALEXIN DEFICIENT4*), which encodes an EDS1‐related lipase‐like protein that forms heterodimers with EDS1 and is required for SA‐mediated defences (Feys *et al*., 2001; Rietz *et al*., 2011), or in *SRFR1*, did not add to *eds1‐2* resistance. Indeed, only genotypes that had a functional *SRFR1* gene together with a functional *EDS1* gene were fully susceptible, suggesting a novel form of cyst nematode resistance in *srfr1* roots.

## Results

### Enhanced resistance of *srfr1‐1* to *S* *. exigua* and *H* *. schachtii*

**Figure 1:**
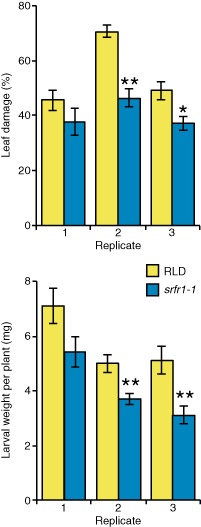
*srfr1‐1* plants are more resistant than RLD wild‐type plants to feeding by *S* *podoptera exigua*. Digitally quantified leaf damage proportions of RLD and *srfr1‐1* (top) and the weight of caterpillars feeding on these plants (bottom) were determined in no‐choice assays. Two first instar larvae confined to a single 4‐week‐old plant were allowed to feed for 7 days. The value shown for a genotype is the average for 26–30 plants. Error bars represent the standard error. Asterisks represent significance as determined by Student's *t*‐test (*P < 0.01, **P < 0.005).

To examine resistance to insect herbivory by *S. exigua*, two first instar larvae were confined to and allowed to feed on a 4‐week‐old plant for 7 days. Each replicate consisted of 26–30 plants per genotype. Leaves of *srfr1‐1* were more resistant than RLD leaves, as shown by significant differences in the proportion of leaf eaten and in the lower mean larval weight per plant in two of three experiments (Fig. 1). In a two‐choice assay, in which *srfr1‐1* and RLD were grown in the same pot and larvae were allowed to select a plant for feeding, larvae showed a significant difference in the choice of RLD leaves over *srfr1‐1* leaves in two of four experiments (Fig. S1, see Supporting Information). In this assay, smaller plants, shorter feeding times and a larger variation in the beginning of feeding time probably contributed to the greater variability.

**Figure 2:**
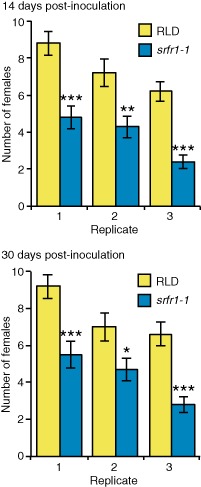
*srfr1‐1* is more resistant than RLD to *H* *eterodera schachtii* infection. Number of fourth‐stage infective juvenile (J4) and adult females per plant at 14 days (top) and 30 days (bottom) post‐inoculation. Error bars represent standard error, and the averages shown represent 20–33 plants for each genotype per replicate. Asterisks indicate significant differences determined by Student's *t*‐test (*P < 0.05, **P < 0.005, ***P < 0.0005).

To evaluate the resistance of *srfr1‐1* and RLD to nematode infection by *H. schachtii*, 14‐day‐old Arabidopsis seedlings (20–33 seedlings of each genotype per replicate) were inoculated with approximately 200 J2s per plant, and the number of fourth‐stage infective juveniles (J4s) and adult female nematodes were determined at 14 and 30 days post‐inoculation. The results showed that *srfr1‐1* was 33%–55% more resistant than RLD and that resistance had occurred by day 14 (Fig. 2). We next determined whether the smaller number of nematodes per *srfr1‐1* plant was a result of decreased penetration. The number of nematodes that had penetrated the roots at 3 days post‐inoculation was similar for both genotypes (Fig. S2, see Supporting Information), indicating that the difference between RLD and *srfr1‐1* resistance to nematode infection most probably results from an altered defence response in *srfr1‐1*.

One explanation for the greater resistance of *srfr1‐1* relative to RLD in our insect feeding and nematode infection experiments and the previously reported constitutively upregulated *PR1*, *PR2* and *PDF1.2* (*PLANT DEFENSIN1.2*) defence gene expression in *srfr1‐1* plants (Kim *et al*., 2009a) could be higher endogenous levels of JA, JA‐Ile and SA in *srfr1‐1* leaves and roots. However, no significant differences in defence hormone levels between RLD and *srfr1‐1* were found in untreated shoot or root tissue (Fig. S3, see Supporting Information). Therefore, we reasoned that the greater resistance to *S. exigua* feeding and *H. schachtii* infection may result from altered sensitivity to defence hormones in *srfr1‐1*, which would manifest itself as changes in constitutive or induced SA pathway or JA pathway gene expression levels in *srfr1‐1*.

### Differential induction of SA‐ and JA‐responsive transcripts in *srfr1‐1* and RLD leaves

We examined *S. exigua*‐induced changes in transcript levels of a subset of SA and JA response marker genes (Fig. S4, see Supporting Information) in local and systemic *srfr1‐1* and RLD leaves using quantitative real‐time PCR (qRT‐PCR). Because the pressure of the cage on the leaves can cause thigmotropic stimuli that may contribute to elevated gene expression (Rehrig *et al*., 2014), we also measured transcript levels in mock‐treated leaves on which cages were applied without insects. At 24 h, *SALICYLIC ACID INDUCTION DEFICIENT2 (SID2)* in the SA pathway and transcript levels of all analysed genes in the JA and JA/ET pathways, except *JASMONATE‐ZIM‐DOMAIN PROTEIN1 (JAZ1)*, showed consistently higher average levels of expression in mock‐treated *srfr1‐1* leaves relative to RLD, although not all differences were supported statistically (Fig. S4). Overall, this indicates a generalized constitutive defence or induction by thigmotropic stimuli in *srfr1‐1*, consistent with the reported constitutive upregulation of SA‐ and JA‐responsive defence genes in *srfr1* plants (Kim *et al*., 2009a).

In size‐matched insect‐damaged leaves at 24 h, there were differences between *srfr1‐1* and RLD in terms of *SID2* levels and JA marker genes (Fig. S4). Transcript levels of genes in the MYC2 branch [*LOX2* (*LIPOXYGENASE2*), *COI1* (*CORONATINE INSENSITIVE1*), *MYC2* and *VSP2* (*VEGETATIVE STORAGE PROTEIN2*)] were higher in *srfr1‐1* than in RLD (Fig. S4). In contrast, *SID2*, *OCTADECANOID‐RESPONSIVE ARABIDOPSIS AP2/ERF59 (ORA59), PDF1.2* and *JAZ1* were expressed to a lower level in insect‐damaged *srfr1‐1* leaves than in RLD (Fig. S4). We did not observe the co‐regulation of *MYC3* with *MYC2* in *srfr1‐1* plants, and *MYC4* expression was very low (Fig. S4). Younger leaves on insect‐eaten plants were also harvested to measure systemically induced gene expression. Induction in *srfr1‐1* of the MYC2 branch of the JA pathway was not as comprehensive in systemic leaves as in insect‐damaged leaves: *LOX2*, *COI1* and *VSP2* transcript levels were higher than in mock‐treated leaves, whereas *MYC2* levels were not (Fig. S4). In systemic leaves of RLD, as in insect‐damaged leaves, the expression of all genes, except *MYC4*, was higher than in mock‐treated leaves, again suggesting generalized defence responses of RLD as a result of insect herbivory (Fig. S4).

**Figure 3:**
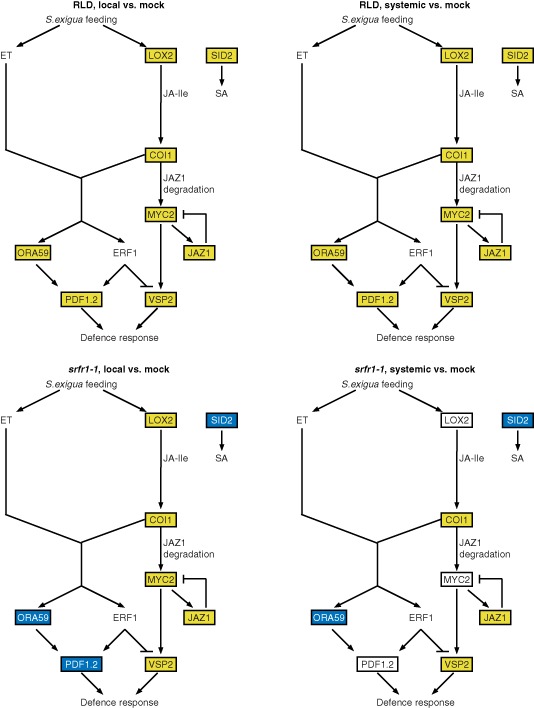
RLD responds to *S* *podoptera exigua* feeding with generalized gene induction, whereas, in *srfr1‐1*, specifically jasmonic acid (JA)‐responsive genes are upregulated. Schematics of simplified JA/ethylene (ET) and salicylic acid (SA) pathways. Marker gene mRNA levels were taken from the data presented in Fig. S4. Genes that were upregulated in both replicates of the indicated pair‐wise comparisons are shown in a yellow box, whereas downregulated genes are shown in a blue box (empty box: no consistent change). See Table S1 for Wilcoxon signed‐rank test statistics.

**Figure 4:**
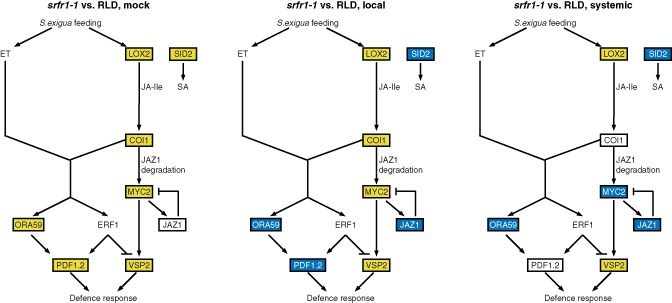
*srfr1‐1* specifically upregulates the MYC2 branch of the jasmonic acid (JA) response pathway on *S* *podoptera exigua* feeding compared with RLD. Schematics of simplified JA/ethylene (ET) and salicylic acid (SA) pathways. Marker gene mRNA levels were taken from the data presented in Fig. S4. Genes that were upregulated in both replicates of the indicated pair‐wise comparisons are shown in a yellow box, whereas downregulated genes are shown in a blue box (empty box: no consistent change). See Table S1 for Wilcoxon signed‐rank test statistics.

These consistent patterns in expression between RLD and *srfr1‐1* responses to herbivory were analysed statistically using Wilcoxon signed rank tests on pair‐wise treatment comparisons of changes in mRNA levels mapped onto a simplified network (Figs 3 and 4; Table S1, see Supporting Information). Comparing transcript levels in RLD insect‐damaged or systemic leaves with those in mock‐treated leaves, the patterns show a statistically significant (*α* < 0.01) generalized herbivory‐induced upregulation of SA‐, JA‐ and JA/ET‐responsive genes (Fig. 3). In contrast, a comparison of levels of gene expression in insect‐damaged or systemic leaves with mock‐treated *srfr1‐1* leaves yielded no statistically significant upregulation over all genes. Instead, the patterns suggest that *srfr1‐1* resistance to *S. exigua* correlates with significant (*α* = 0.01) herbivory‐induced upregulation specifically of MYC2 branch JA‐responsive genes, leading to *VSP2* upregulation (‘MYC2 branch’ genes) in insect‐damaged leaves (Fig. 3). This contrast is more easily discerned when expression levels in different leaf tissues are compared between RLD and *srfr1‐1* (Fig. 4). In these analyses, all genes as a group are significantly higher (*α* < 0.01) in mock‐treated leaves of *srfr1‐1* compared with RLD. In contrast, insect‐damaged *srfr1‐1* leaves show a significant (*α* = 0.01) upregulation of MYC2 branch genes, concomitant with suppression of remaining SA‐ and JA/ET‐responsive genes compared with RLD (Fig. 4). In systemic *srfr1‐1* leaves, upregulation of MYC2 branch genes is not observed (*α* > 0.1), whereas the suppression of the remaining genes is still significant (*α* = 0.02; Fig. 4, Table S1).

Results at later time points suggest that insect feeding‐induced changes in transcript levels are not likely to persist after 24 h. In insect‐damaged and systemic leaves of both genotypes, *LOX2* and *VSP2* expression was greatly reduced between 24 and 48 h after treatment (Figs S4 and S5, see Supporting Information). Although *COI1* continued to be expressed at a level similar to that at 24 h, there was no difference between *srfr1‐1* and RLD. Only the *PDF1.2* transcript was higher in *srfr1‐1* systemic leaves than in RLD at 48 h (Figs S4 and S5).

Thus, as summarized in Figs 3 and 4, the SA, JA and JA/ET defence pathways appear to be primed in *srfr1‐1* relative to RLD. In contrast, 24 h after insect feeding, *srfr1‐1* showed specifically increased transcript levels in the pathway most likely to be effective against *S. exigua*, the MYC2 branch of the JA pathway, whereas a generalized induction of defence pathways was seen in RLD.

### Differential induction of SA‐ and JA‐responsive transcripts in *srfr1‐1* and RLD roots and leaves

**Figure 5:**
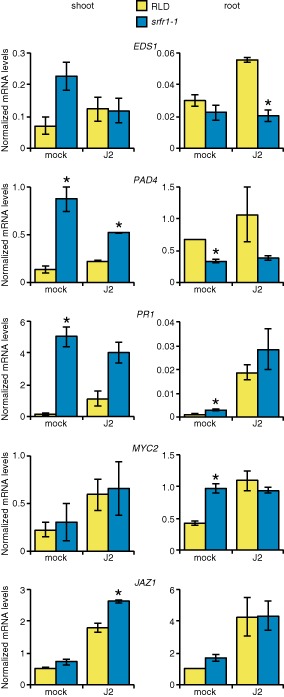
Downregulation of *ENHANC EDS* *1* in *srfr1‐1* roots relative to RLD after *H* *eterodera schachtii* infection. Transcript levels of the indicated salicylic acid (SA) and jasmonic acid (JA) pathway genes in shoot (left) and root (right) tissue of RLD and *srfr1‐1* at 3 days post‐inoculation of roots with agarose (mock) or *H* *. schachtii* (second‐stage juveniles, J2s) as measured by quantitative real‐time PCR (qRT‐PCR) with normalization using *SAND* gene (At2g28390) mRNA levels as an internal standard. Values represent averages from two biological replicates, with error bars showing the standard error. Asterisks denote significant differences between *srfr1‐1* and RLD expression levels in a treatment (mock or J2) as determined by Student's *t*‐test (*P* < 0.05).

To evaluate the contribution of the SA and JA signalling pathways to the increased resistance of *srfr1‐1* to *H. schachtii*, transcript levels of SA‐responsive (*EDS1*, *PAD4* and *PR1*) and JA‐responsive (*MYC2* and *JAZ1*) genes were determined in 14‐day‐old Arabidopsis seedlings at 3 days post‐infection and compared with transcript levels in mock‐treated seedlings. In seedling shoots of mock‐treated *srfr1‐1*, levels of the SA‐responsive transcripts *PAD4* and *PR1* were elevated compared with RLD, whereas no differences were evident for the JA‐responsive transcripts (Fig. 5). After nematode infection, levels of SA‐responsive transcripts were reduced in *srfr1‐1* seedling shoots and remained low in RLD, suggesting suppression of systemic SA‐mediated defence and resulting in little difference between the two, except for *PAD4* (Fig. 5). JA‐responsive transcripts increased in shoots of both RLD and *srfr1‐1* (Fig. 5). Thus, *srfr1‐1* shoots appeared to be primed in the SA pathway relative to RLD; however, soon after nematode infection, this difference was largely lost.

In roots, we observed that, in both genotypes, the levels of *EDS1* and *PR1* transcripts were much lower than systemic shoot levels (Fig. 5). Lower *PR1* expression in roots than shoots has been reported previously (Seo *et al*., 2008). In mock‐treated roots, there were no clear patterns in the comparison of mRNA levels of SA‐responsive and JA‐responsive genes in *srfr1‐1* and RLD (Fig. 5). The transcript level of *PAD4* was higher in RLD, whereas *MYC2* and *PR1* were higher in *srfr1‐1*, but there was little difference in *EDS1* and *JAZ1* transcript levels (Fig. 5). Comparing mock treatment with nematode infection within a genotype suggested that both RLD and *srfr1‐1* increased or maintained high levels of *MYC2* and *JAZ1* transcripts after infection with *H. schachtii* (Fig. 5), resulting in no difference between *srfr1‐1* and RLD after J2 treatment. In the SA pathway, both genotypes increased *PR1* transcript levels and maintained *PAD4* transcript levels, whereas *EDS1* transcript levels increased only in RLD (Fig. 5). Thus, after infection with *H. schachtii* J2s, the lower *EDS1* transcript level in *srfr1‐1* roots than in RLD suggests that increased *EDS1* transcript levels in roots may be associated with increased susceptibility to nematodes.

**Figure 6:**
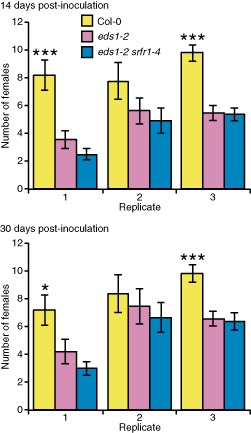
*eds1‐2* plants are more resistant than Col‐0 plants to nematode infection. The number of fourth‐stage juvenile (J4) and adult females per plant at 14 days (top) and 30 days (bottom) post‐inoculation obtained from 22–35 samples per genotype. Error bars represent the standard error. Asterisks indicate a significant difference determined by Student's *t*‐test (*P < 0.05, ***P < 0.0005).

**Figure 7:**
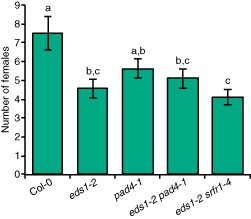
*EDS* *1* and *SRFR* *1*, but not *PAD* *4*, contribute to nematode susceptibility. The number of fourth‐stage juvenile (J4) and adult females per plant at 30 days post‐inoculation obtained from 20–30 samples per genotype. Error bars represent standard error, with different letters denoting significant differences (*P* < 0.05) as determined by Student's *t*‐test. This experiment was repeated once with similar results.

To test the significance of this surprising finding, we performed infection assays with the corresponding mutants in the Col‐0 background, which is also susceptible to *H. schachtii*. Because *srfr1* mutants in the Col‐0 background are severely stunted (Kim *et al*., 2010; Li *et al*., 2010), they could not be used for cyst nematode infection assays; however, growth of the double mutant *eds1‐2 srfr1‐4* is normal (Bhattacharjee *et al*., 2011). No difference in root morphology was observed in all tested genotypes. Both *eds1‐2* and *eds1‐2 srfr1‐4* showed significantly enhanced resistance relative to wild‐type Col‐0 at 14 and 30 days post‐inoculation in two of three replications (Fig. 6), confirming that a functional *EDS1* gene correlates with increased susceptibility. The enhanced resistance of *eds1‐2 srfr1‐4* and *eds1‐2* was not significantly different, indicating that *srfr1‐4* and *eds1‐2* do not have additive effects. In another set of experiments that included the *pad4‐1* mutant, enhanced resistance was found in all tested *eds1‐2* single and double mutants relative to wild‐type Col‐0 (Fig. 7), again indicating that functional *EDS1* promotes parasitism by *H. schachtii*. The absence of *PAD4* did not enhance resistance in *eds1‐2 pad4‐1* double mutants relative to *eds1‐2*, and the susceptibility of *pad4‐1* to *H. schachtii* was not statistically significantly different from that of Col‐0 (Fig. 7). This suggests that *PAD4* does not play a role in susceptibility or resistance to this nematode. Surprisingly, therefore, within these tested genotypes, maximal susceptibility was observed when plants possessed both a functional *SRFR1* and *EDS1* gene.

## Discussion

Here, we have shown that mutations in *SRFR1* lead to reduced leaf herbivory by *S*. *exigua* and to reduced development of *H*. *schachtii*. We have also shown that there were marked differences in leaf and root transcript level patterns between genotypes and in the responses to insect feeding and nematode parasitism. Insect‐damaged *srfr1‐1* leaves 24 h after feeding showed specifically increased JA‐responsive and concomitantly suppressed SA‐ and JA/ET‐responsive transcript levels. The importance of the JA‐mediated response to wounding and herbivory has been well documented in transcriptome analyses and in studies with mutants impaired in the perception or synthesis of JA (Reymond *et al*., 2004; Song *et al*., 2014; Verhage *et al*., 2011; Wu and Baldwin, 2010). Consistent with our gene expression results, in a recent feeding study, *Pieris rapae* and *S. exigua* larvae gained most weight when feeding on *myc2 myc3 myc4* mutants, intermediate weight on Col‐0 and least weight on *ein3 eil1* mutants (Song *et al*., 2014). Correspondingly, the application of methyl jasmonate resulted in reduced levels of *VSP2* transcripts in *myc2 myc3 myc4* plants relative to Col‐0, but increased levels in *ein3 eil1* plants.

In contrast with *srfr1‐1*, the generalized increase in all analysed defence marker transcripts in RLD and the pronounced increase in the *JAZ1* transcript, which can inhibit JA pathway induction, correlated with greater leaf damage by *S. exigua*. In a recent report, *MYC2*, *ORA59*, *PDF1.2*, *PR3* and *PR4* transcript levels were elevated in Col‐0 local leaves 6 h after *S. exigua* feeding, suggesting a similar generalized upregulated defence in another susceptible accession (Rehrig *et al*., 2014). In addition, JA, JA‐Ile and ET were increased by 30 min and remained higher 6 h after initiation of *S. exigua* feeding, confirming that the larvae activated both JA and ET pathways. In that study, transcript levels had decreased by 24 h, except for *PR3*, perhaps because the feeding time was shorter and there was less damage. Nevertheless, transcript levels of 23 transcription factors in 10 classes were elevated in the local leaf 24 h after feeding (Rehrig *et al*., 2014).

Although *SRFR1* has a well‐described function in effector‐triggered immunity as a negative regulator of TNL‐mediated EDS1‐dependent resistance (Gassmann and Bhattacharjee, 2012), we consider it unlikely that alteration of this sector of the defence network can explain the increased resistance to insect feeding displayed by *srfr1‐1*. Instead, *srfr1‐1* leaves appear to respond more strongly in either the JA or SA pathway, depending on the stimulus, placing *SRFR1* as a negative regulator at a cross‐point between these two pathways. We propose that the recently described interaction of SRFR1 with class I TCP transcription factors (Kim *et al*., 2014) may form the basis for these observations. SRFR1 interacts most strongly with TCP8, TCP14 and TCP15, and less strongly with TCP20, TCP22 and TCP23. Because a *tcp8 tcp14 tcp15* triple mutant was compromised in effector‐triggered immunity to DC3000 expressing the effectors AvrRps4, HopA1, AvrRpm1 or AvrRpt2, it appears that these TCPs positively regulate general defence genes active against DC3000, and that SRFR1 functions as a co‐repressor by interacting with TCPs (Bhattacharjee *et al*., 2013; Kim *et al*., 2014).

Interestingly, the class I TCPs TCP20 and TCP9 have been reported recently to negatively regulate *LOX2* expression in developing leaves, whereas the class II TCP TCP4 induces *LOX2* (Danisman *et al*., 2012; Schommer *et al*., 2008). If this repressive function depends on SRFR1 as a co‐repressor, one would predict an upregulation of *LOX2* in *srfr1* mutants. This upregulation may be partly constitutive, but mainly more pronounced after a stimulus, which matches our observations. A connection of TCPs to insect defences would underscore the emerging importance of TCPs in plant biotic stress responses to a wide range of organisms (Kim *et al*., 2014; Mukhtar *et al*., 2011; Sugio *et al*., 2011; Weßling *et al*., 2014). Ultimately, induction of *LOX2* and the JA pathway would result in the accumulation of VSP2 (Berger *et al*., 2002; Liu *et al*., 2005; Reymond *et al*., 2004) and probably other insect deterrents, such as glucosinolates (Mewis *et al*., 2005; Rehrig *et al*., 2014; Schweizer *et al*., 2013; Textor and Gershenzon, 2009). Alternatively, SRFR1's role in herbivory responses may depend on its interaction with the receptor co‐chaperone constituent SGT1b (Li *et al*., 2010), which, together with heat shock proteins HSP90 and HSP70, stabilizes the JA receptor COI1 (Zhang *et al*., 2015). Consistent with this, SGT1 in *Nicotiana attenuata* has been shown to positively regulate JA accumulation and resistance to *Manduca sexta*, a specialist herbivore (Meldau *et al*., 2011).

Comparing the results obtained from leaf bacteria and insects with those from nematodes feeding on RLD and *srfr1‐1* indicates that defence signalling pathways in shoots and roots differ. Because *H. schachtii* parasitism was inhibited by SA treatment of wild‐type Col‐0, was increased in SA‐deficient *NahG*‐expressing plants and the *sid2‐1* mutant (Wubben *et al*., 2008), and was reduced by overexpression of *PR1* (Hamamouch *et al*., 2011), we expected increased parasitism in *eds1‐2* plants. However, in our experiments, increased levels of *EDS1* transcript in RLD relative to *srfr1‐1* suggested that induction of the SA pathway correlated with susceptibility. Mutation of either *SRFR1* or *EDS1* led to similar levels of resistance, and the simultaneous presence of *EDS1* and *SRFR1* appeared to correlate with increased susceptibility to nematode parasitism, suggesting a novel form of resistance to nematodes or a different regulation of the SA pathway by EDS1 in roots.

It is possible that EDS1 constitutes an important virulence target for a nematode effector. Because the *eds1‐2* mutant was more resistant to *H. schachtii*, the function of such an effector would probably not consist of the blocking or degradation of EDS1, but rather of the hyperactivation of EDS1's negative effect on root nematode resistance. In this interaction, EDS1 in roots does not appear to occupy as central and positive a role in plant resistance and SA pathway regulation as in shoots. It was shown that SA is required for *Mi‐1*‐mediated resistance to root‐knot nematodes; in contrast, a functional JA pathway was required for nematode parasitism, perhaps via antagonism of the SA pathway (Bhattarai *et al*., 2008). Future research will therefore need to examine whether, in Arabidopsis roots, the SA pathway is still fully active against *H. schachtii* in the absence of *EDS1*.

*Heterodera* effectors that impact plant processes, such as signalling, defence and cell wall remodelling, are beginning to be identified (Hewezi and Baum, 2013; Mitchum *et al*., 2013). Nematode infection is a complex process requiring cell cycle alteration, reprogramming of root cells that form the feeding site, and altered plant metabolism. A time course study of infection between 3 days post‐infection, when penetration was assayed, and day 14, when resistance was observed, could narrow down when the responses in *srfr1‐1* and *eds1‐2* roots begin to differ from those in wild‐type plants and uncover the affected processes. In addition, further studies need to determine whether full susceptibility requires *EDS1* and *SRFR1* expression locally (in the developing syncytium) or in the surrounding root tissue.

In summary, the results here expand on and provide first insights into the heightened resistance of *srfr1‐1* to a wide spectrum of biotic stresses, and demonstrate the value of using multiple stresses to better understand gene function. A more complete analysis of gene expression and defence hormone level changes in response to *Pseudomonas syringae*, *S. exigua* and *H. schachtii* will provide insights into the molecular mechanism of system‐wide defence regulation by SRFR1. In addition, the effects of mutation of *SRFR1* on the yield and resistance of food crops need to be tested to determine whether decreasing expression of this regulator could be an asset in disease and pest control.

## Experimental Procedures

### Insect bioassays and plant growth

Eggs of *S. exigua* were obtained from Benzon Research (Carlisle, PA, USA) and larvae reared on artificial diet (Bioserv, Frenchtown, NJ, USA). First instar caterpillars were transferred to RLD and *srfr1‐1* plants 1 day before the experiments for acclimation as described by Rehrig *et al*. (2014). All seeds were germinated in half‐strength Murashige and Skoog (MS) medium containing 1% agar and sown into 6 × 5‐cm^2^ pots containing sterile Metromix 200 soil (Sun Gro Horticulture Ltd., Agawam, MA, USA) and grown in chambers at 22 °C, 65% ± 5% relative humidity and light intensity of 200 μmol/m^2^/s with a short‐day photoperiod (8 h light : 16 h dark), as described by Rehrig *et al*. (2014). Each of three bioreplications consisted of 30 4–5‐week‐old plants of each genotype and 10 mock‐treated plants of each genotype.

To estimate plant performance in response to herbivore feeding, no‐choice assays and two‐choice assays were conducted. In the no‐choice assay, RLD and *srfr1‐1* were grown in separate pots, and two first‐instar larvae were fed for 1 week on each plant. The plant rosettes were caged by transparent Mylar cylinders (diameter, 5 cm; height, 9 cm) with fine mesh gauze tops (<0.01‐mm mesh) to maintain air exchange. In the two‐choice assay, both RLD and *srfr1‐1* were grown in the same pot caged by a similar cylinder, and one second‐instar caterpillar was placed between the two plants for free selection during a 16‐h feeding period. Camel hair brushes were used to catch and move the caterpillars. Mock treatments were caged plants without a caterpillar, and all plants were returned to the growth chamber during the caterpillar feeding time.

To investigate early gene expression events, one third‐instar caterpillar was forced to feed on a single seventh fully expanded mature leaf that had half its area caged by a clip with fine mesh gauze (<0.01‐mm mesh). The caterpillar was allowed to feed for 3–5 h until 40%–50% of the leaf area was removed. Cages without insects were placed on size‐matched leaves on mock‐treated plants. Once sufficient damage was achieved, the caterpillar was removed and the plant was returned to the growth chamber. Caterpillar‐damaged leaves and two systemic leaves, younger leaves on both sides of the damaged leaf, were harvested for gene expression analysis at 24 and 48 h after feeding. Caged leaves on mock‐treated plants were harvested at the same time.

### Quantification of eaten leaf area and caterpillar weight measurement

Photographs of RLD and *srfr1‐1* leaves before feeding (0 days) and after feeding (7 days) were taken with a 10‐megapixel Canon (Melville, NY, USA) Rebel digital camera in a customized stand. They were analysed with a computer algorithm that automatically corrects image size, setting the green areas to 10 000 pixels/cm^2^, and then masks the leaf images to calculate the pixels of leaf tissues (Green *et al*., 2012). Untreated plants were used to determine the increases in leaf area resulting from growth, and the growth rates of each genotype were factored into calculations of the final area of leaf tissue removed by a caterpillar.

The caterpillar weight at the beginning of a feeding assay was assumed to be zero. After feeding, caterpillars in the no‐choice assay were weighed with an analytical balance (Mettler Toledo, LLC, OH, USA) and the average growth during a 1‐week feeding period was calculated. The 16‐h feeding period in the choice assay was too short for meaningful measurement of caterpillar growth.

### Plant material and growth conditions for nematode assays

All Arabidopsis plants were grown from surface‐sterilized seed planted on modified Knops nutrient medium, pH 6.4, with 0.8% Daishin agar (Brunschwig Chemie, Amsterdam, the Netherlands) (Sijmons *et al*., 1991) on 12‐well plates or 9‐cm^2^ vertical plates (Falcon Brand; BD, Franklin Lakes, NJ, USA) in a growth chamber under 16‐h light : 8‐h dark at 25 °C (Replogle *et al*., 2013). Using *eds1‐2* plants as a recipient, *eds1‐2 srfr1‐4* and *eds1‐2 pad4‐1* double knockout plants were generated and genotyped by polymerase chain reaction (PCR) with the primers, as described previously (Bartsch *et al*., 2006; Kim *et al*., 2010; Ng *et al*., 2011).

### Cyst nematode infection assays

*Heterodera schachtii* eggs were collected and surface sterilized in 0.02% sodium azide for 20 min, washed with tap water and set up to hatch at 27 °C on a modified Baermann pan for 2–3 days in antibiotic solution containing 1.5 μg/mL gentamicin and 0.05 μg/mL nystatin. Freshly hatched infective J2s were collected and surface sterilized for 8 min in solution containing 0.04% mercuric chloride, 0.04% sodium azide and 0.002% Triton X‐100, washed four times in sterile water, resuspended in sterile 0.1% agarose with rotation for 60 min and diluted to a concentration of approximately 200 J2s/25 μL resuspended in sterile 0.1% agarose (Replogle *et al*., 2013). Each 14‐day‐old Arabidopsis seedling was inoculated with 25 μL of this J2 suspension. The numbers of J4 and adult females that developed on each plant by 14 and 30 days post‐inoculation were counted under a dissecting microscope. The experimental design for the assessment of mutant susceptibility to *H. schachtii* employed randomly arranged mutant and wild‐type seeds within 12‐well plates, with one seed per well. Each individual mutant was analysed in at least three independent experiments with a minimum of 20 plants per experiment. Data were analysed using Student's *t*‐test.

### Penetration assay

Fourteen‐day‐old RLD and *srfr1‐1* plants grown on 12‐well plates were each inoculated with 25 μL of agarose containing approximately 200 J2s. Acid fuchsin staining was performed at 3 days post‐inoculation as described by Ithal *et al*. (2007) with some modifications. The whole intact root in each well was gently separated from agarose medium, and transferred to a new 12‐well plate with water to wash out agarose. The roots in each well were incubated in 10% bleach for 7 min, followed by 20‐min incubation in tap water. Fifty millilitres of water were brought to the boil in a glass beaker to which 1 mL of acid fuchsin solution (0.35 g acid fuchsin in 25 mL of acetic acid) was added. Three millilitres of boiled acid solution were added to each root twice, 2 min apart. The roots were then incubated in acid fuchsin for 7–10 min. Root segments were rinsed with water and stored in 1.5‐mL Eppendorf tubes containing 100% ethanol. Before observations under a dissecting microscope, the roots were briefly rinsed in water and placed on a slide containing 50% glycerine. Photographs were taken using an Olympus (Center Valley, PA, USA) camera.

### RNA extraction and qRT‐PCR

For insects, the transcript abundance of investigated genes was analysed by qRT‐PCR performed on RNA samples that were generated from Arabidopsis leaf tissue with or without *S. exigua* treatments. For nematodes, plants were grown on vertical plates containing modified Knop's medium, with about 10 plants per plate. Each 8‐day‐old seedling was inoculated with 25 μL of agarose containing about 200 J2s for infected samples or no J2s for mock‐treated samples. At 3 days post‐inoculation, root and shoot tissues from a minimum of 10 plants per genotype were collected.

Total RNA was extracted using Trizol reagent (Invitrogen, Carlsbad, CA, USA) and treated with Turbo DNase (Ambion, Austin, TX, USA). For qRT‐PCR, first‐strand cDNA was synthesized from 2 μg of total RNA using an oligo(dT)15 primer and Moloney murine leukaemia virus (MMLV) reverse transcriptase (Promega, Madison, WI, USA) according to the manufacturer's instructions. qRT‐PCR was performed using an ABI 7500 system and a SYBR Green qPCR Master Mix (Applied Biosystems, Warrington, UK). For insect experiments, a reaction volume of 20 μL was used with the following cycling conditions: 50 °C for 2 min, 95 °C for 10 min, and 40 cycles of 95 °C for 15 s and 59 °C for 1 min. For nematode experiments, a reaction volume of 10 μL was used with the following cycling conditions: 50 °C for 2 min, 95 °C for 10 min, and 40 cycles of 95 °C for 15 s and 65 °C for 1 min, followed by a dissociation step of 95 °C for 15 s. For a given cDNA sample, reactions were run in duplicate or triplicate (technical replicates), each for two biological replicates.

Primers used in qRT‐PCR experiments are listed in Table S2 (see Supporting Information). Transcript levels of a target gene were normalized using the *SAND* gene (Czechowski *et al*., 2005). This gene showed no significant transcript level differences between mock‐treated and wounded plants (Rehrig *et al*., 2011). In the present experiments, the cycle threshold (Ct) values for *SAND* were similar between mock‐treated and nematode‐infected plants in roots and shoots. Quantitative RT‐PCR data were analysed as described previously (Kwon *et al*., 2009). PCR efficiency was calculated using linear regression in the LinRegPCR program (Ramakers *et al*., 2003), and was determined to be >1.68 for all samples with *R* ^2^ > 0.9975. The Ct value for *SAND* was subtracted from the Ct value for target genes. Expression values (Exp) for target genes were calculated as: Exp = (PCR efficiency)^−ΔCt^.

For insect experiments, Wilcoxon signed‐rank tests (Sokal and Rohlf, 1995) were performed on groups of genes to analyse the statistical significance of differences in pathway regulations between two samples. For example, to compare expression levels in RLD insect‐damaged leaves with those in mock‐treated leaves, the ratio of expression values (RLD local/RLD mock) of a given gene from a given biological replicate was determined and log_2_‐transformed. Absolute log_2_‐transformed values of groups of genes from both biological replicates of the same treatment were ranked from lowest to highest values. The test statistics *W^+^* and *W* ^−^ were calculated by adding rank values with positive and negative signs, respectively, and compared with a table of critical *W* values. For nematode experiments, differences in expression levels between RLD and *srfr1‐1* were analysed using Student's *t*‐test, with *P* < 0.05 used as a threshold for statistical significance.

### JA, JA‐Ile and SA quantification

SA, JA and JA‐Ile levels were measured in untreated leaf and root tissues of RLD and *srfr1‐1*. Leaf tissue was harvested from plants grown as described for insect feeding experiments, and root tissue from seedlings as grown for nematode infection assays. SA, JA and JA‐Ile were extracted and measured by ultra performance liquid chromatography‐electrospray ionization‐tandem mass spectrometry (UPLC‐ESI‐MS/MS) as described previously (Chung *et al*., 2008; Koo *et al*., 2009; Smith *et al*., 2014).

### Statistical analyses

All statistical analyses were performed using the program Excel (Microsoft, Redmond, WA, USA).

### Accession numbers

*COI1* (At2g39940), *EDS1* (At3g48090), *JAZ1* (At1g19180), *LOX2* (At3g45140), *MYC2* (At1g32640), *MYC3* (At5g46760), *ORA59* (At1g06160), *PAD4* (At3g52430), *PDF1.2* (At5g44420), *PR1* (At2g14610), *SID2* (At1g74710), *SRFR1* (At4g37460), *VSP2* (At5g24770).

## Supporting information


**Fig. S1** *Spodoptera exigua* larvae tend to prefer RLD over *srfr1‐1* in a two‐choice assay. Single RLD and *srfr1‐1* plants were grown in the same pot and *S. exigua* was allowed to feed for 16 h when plants were 3 weeks old. The digitally quantified leaf area eaten (%) was determined for each plant, and damage on *srfr1‐1* was subtracted from that on RLD for each pair. Positive values indicate more damage on RLD, and negative values indicate more damage on *srfr1‐1*. Differences were statistically significant (*P* < 0.01) by the Wilcoxon signed rank test in two of four replicates.
**Fig. S2** *Heterodera schachtii* penetration assay in RLD and *srfr1‐1* roots. A minimum of 20 infected roots of 14‐day‐old seedlings in 12‐well plates were stained with acid fuchsin at 3 days post‐inoculation. No statistically significant difference between RLD and *srfr1‐1* was detected at *P* < 0.05 as analysed by Student's *t*‐test.
**Fig. S3** Endogenous defence hormone levels in RLD and *srfr1‐1* do not differ. Total resting‐state salicylic acid (SA) (top), jasmonic acid (JA) (middle) and JA‐Ile (JA conjugated with the amino acid isoleucine) (bottom) levels in leaf tissue of RLD and mutant *srfr1‐1* soil‐grown plants (left) or roots of plate‐grown seedlings (right) were quantified by ultra performance liquid chromatography‐electrospray ionization‐tandem mass spectrometry (UPLC‐ESI‐MS/MS). Tissue was harvested at the same stage as used for insect feeding and nematode infection experiments, respectively. Error bars represent standard errors of three to four samples. There were no significant differences between RLD and *srfr1‐1* as determined by Student's *t*‐test (*P* > 0.05). Similar results were found in a second experiment.
**Fig. S4** Differences in jasmonic acid/ethylene (JA/ET) and salicylic acid (SA) pathway gene regulation in *srfr1‐1* and RLD in response to *Spodoptera exigua* feeding. mRNA levels of the indicated SA and JA/ET pathway genes in *Arabidopsis* leaf tissue were determined 24 h after *S. exigua* feeding in mock‐treated (mock), insect‐damaged (local) and systemic (systemic) leaves. Expression levels were quantified by quantitative real‐time PCR (qRT‐PCR) and normalized using *SAND* gene (At2g28390) mRNA levels as an internal standard. Values represent averages from two biological replicates, with error bars showing the standard error.
**Fig. S5** Dampened mRNA level changes 48 h after *S. exigua* feeding. mRNA levels of the indicated JA/ET pathway genes in *Arabidopsis* leaf tissue were determined in mock‐treated (mock), insect‐damaged (local) and systemic (systemic) leaves. Expression levels were quantified by quantitative real‐time PCR (qRT‐PCR) and normalized using *SAND* gene (At2g28390) mRNA levels as an internal standard. Values represent averages from two biological replicates, with error bars showing the standard error. For ease of comparison, the same scales were used for the genes shown in both Figs 3 and 4.
**Table S1** *W* test statistic values for Wilcoxon signed‐rank tests on the data in Figs 3 and 4.
**Table S2** Forward (F) and reverse (R) qRT‐PCR primers.
