# Supplementary material for: The Arabidopsis immune regulator SRFR 1 dampens defences against herbivory by S podoptera exigua and parasitism by H eterodera schachtii
Source: Mol Plant Pathol. 2015 Nov 6;17(4):588–600. doi: 10.1111/mpp.12304 (PMC6638418; doi:10.1111/mpp.12304)
Supplement: Supplementary file 1 — Fig. S1 Spodoptera exigua larvae tend to prefer RLD over srfr1‐1 in a two‐choice assay. Single RLD and srfr1‐1 plants were grown in the same pot and S. exigua was allowed to feed for 16 h when plants were 3 weeks old. The digitally quantified leaf area eaten (%) was determined for each plant, and damage on srfr1‐1 was subtracted from that on RLD for each pair. Positive values indicate more damage on RLD, and negative values indicate more damage on srfr1‐1. Differences were statistically significant (P < 0.01) by the Wilcoxon signed rank test in two of four replicates. Fig. S2 Heterodera schachtii penetration assay in RLD and srfr1‐1 roots. A minimum of 20 infected roots of 14‐day‐old seedlings in 12‐well plates were stained with acid fuchsin at 3 days post‐inoculation. No statistically significant difference between RLD and srfr1‐1 was detected at P < 0.05 as analysed by Student's t‐test. Fig. S3 Endogenous defence hormone levels in RLD and srfr1‐1 do not differ. Total resting‐state salicylic acid (SA) (top), jasmonic acid (JA) (middle) and JA‐Ile (JA conjugated with the amino acid isoleucine) (bottom) levels in leaf tissue of RLD and mutant srfr1‐1 soil‐grown plants (left) or roots of plate‐grown seedlings (right) were quantified by ultra performance liquid chromatography‐electrospray ionization‐tandem mass spectrometry (UPLC‐ESI‐MS/MS). Tissue was harvested at the same stage as used for insect feeding and nematode infection experiments, respectively. Error bars represent standard errors of three to four samples. There were no significant differences between RLD and srfr1‐1 as determined by Student's t‐test (P > 0.05). Similar results were found in a second experiment. Fig. S4 Differences in jasmonic acid/ethylene (JA/ET) and salicylic acid (SA) pathway gene regulation in srfr1‐1 and RLD in response to Spodoptera exigua feeding. mRNA levels of the indicated SA and JA/ET pathway genes in Arabidopsis leaf tissue were determined 24 h after S. exigua feeding in mock‐tr [file MPP-17-588-s001.zip › Table S2_proofs.docx]

**Table S2.** Forward (F) and reverse (R) qRT-PCR primers.

| qRT-PCR primers | AGI number | Sequence (5’-3’) |
| --- | --- | --- |
| *SAND*  *COI1*  *LOX2*  *VSP2*  *MYC2*  *MYC3*  *MYC4*  *PDF1.2*  *JAZ1*  *ORA59* | AT2G28390  AT2G39940  AT3G45140  AT5G24770  AT1G32640  [AT5G46760.1](http://www.arabidopsis.org/servlets/TairObject?id=136604&type=gene)  AT4G17880  AT5G44420  AT1G19180  AT1G06160 | F- AACTCTATGCAGCATTTGATCCACT  R- TGATTGCATATCTTTATCGCCATC  F-GTGAGCGAGCAATCGCTGCAGC  R-CAGCTCGATGTTCCAGTACGGTC  F- CAGGCGACCCTAGTGATGGT  R- TTGATAACAGGCTCATTGGC  F- GATACGGAACAGAGAAGACC  R- AGCTTCGAGATTGTCGAGAG  F- CCCGGGAATGACTGATTACCGGCTACA  R- CCCGGGTTAACCGATTTTTGAAATCAA  F- TGTTGAAGCAGAGAGGCAGA  R- CTCCGAGAAGCGAAGCTTTA  F- AGGAGCAAACGAGAACTGGA  R- CCATCTCCCCAACCTAACAA  F- AAGTTGTGCGAGAAGCCAAG  R- CCATGTTTGGCTCCTTCAAG  F- GAATTCATGTCGAGTTCTATGGAATGTTCT  R- CTCGAGTCATATTTCAGCTGCTAAACCGAG  F-GGGATAAGAGTGTGGCTTGGGACA  R-TGAGTACTGCGAGGCTGCCTTT |

**Table S1** continued

| qRT-PCR primers | AGI Number | Sequence (5'-3') |
| --- | --- | --- |
| *PR1*  *EDS1*  *PAD4*  *SID2* | AT2G14610  [AT3G48090](http://www.arabidopsis.org/servlets/TairObject?id=39706&type=locus)  AT3G52430  AT1G74710 | F-CCCTCGAAAGCTCAAGATAG  R-GTTCACATAATTCCCACGAGG  F-GACGGGGAAGTAGATGAGAAG  R-TCATCCATCATACGCTCACG  F-GAGGAGATCTTTGTTACGGG  R-TCGCCTCCCACACACTATAA  F-GCAACA ACATCTCTACAGGCG  R-AGAACCCCTTATCCCCCATA |
